# Family cultural capital predicts student cognitive performance: The mediating role of student academic engagement and academic self-efficacy in a comparative cross-national context

**DOI:** 10.1371/journal.pone.0329770

**Published:** 2025-10-30

**Authors:** Denis Djekourmane, Yang Zhang, Ming Li, Zewen Cai

**Affiliations:** 1 Faculty of education, Shaanxi Normal University, Xi’an, Shaanxi Province, China; 2 School of education, Central China Normal University, Wuhan, Hubei Province, China; The Open University of Israel, ISRAEL

## Abstract

This study examines the relationships between family cultural capital and student cognitive performance, emphasizing the mediating effects of psychological and behavioral factors labeled student academic engagement and academic self-efficacy. Leveraging the PISA 2022 data, comprising 378,306 respondents from 33 OECD countries and 38 non-OECD countries/economies, we employed Partial Least Square-Structural Equation Modeling (PLS-SEM) analysis to test the hypothesized pathways and compare the relationships. The results revealed that family cultural capital has positive direct impacts on student cognitive performance, student academic engagement, and academic self-efficacy. Moreover, student academic engagement and academic self-efficacy are also positively associated with student cognitive performance. However, when comparing the results, all the direct effect sizes vary between OECD and non-OECD countries. Furthermore, student academic engagement and academic self-efficacy partially mediated the relationships, albeit with lower effects in OECD countries. The findings support the theory that cultural capital fosters student cognitive skills, also highlighting the crucial role that psychological and behavioral factors play in mediating these relationships and considering the differences between countries for theoretical and practical implications.

## Introduction

For decades, numerous studies on cognitive psychology have been conducted and provided important insights about cognitive performance [1,2]. People’s cognitive abilities, known as the process and the function of psychological components for a human’s brain to process, store, logically reason, and extract information within the surrounded environment, are considered a key motivator for a human’s success [3,4]. In education, students’ cognitive performance focuses on their dealing with information, capacity to memorize, ability and skills in logical reasoning and analytical thinking when solving problems, as well as how they creatively transform the crystallized knowledge [5]. Researchers have demonstrated a special interest in enhancing student cognitive performance, perceiving it as a complex human intelligence that manifests through their literacy and numeracy skills [1,6].

The past studies on student cognitive achievement have shown that family cultural capital, which includes objective, institutionalized, and embedded variables, can also predict student cognitive outcomes [7–9]. Studies have demonstrated that students’ family environment, which provides both material and non-material cultural resources, directly and indirectly improves their school grades and cognitive skills [10,11]. For instance, Andersen et al. [12] found that the cultural capital rooted in the family profoundly influences the development of students’ reading abilities and their academic achievement.

In connection with this, scholars have emphasized the crucial role that students’ academic engagement, defined as the degree of attention, emotional involvement, and cognitive processes that students exhibit when dealing with academic-related issues, plays in their cognitive performance [13,14]. Acosta-Gonzaga [15] and Qureshi et al. [16] discovered that students’ self-esteem influences their emotional and behavioral motivation, which in turn leads to academic engagement as well as student metacognitive engagement, which in turn drives cognitive performance.

Self-efficacy is an important psychological and non-cognitive variable that affects motivation and interest at the individual level and is used by researchers to predict cognitive performance [17]. Introduced in psycho-educational studies by Bandura, self-efficacy is considered an individual’s confidence about their capabilities to effectively perform some tasks [18]. Student academic self-efficacy empowers learners to make the right choices in an effective learning environment and engage through persistence, resilience, and positive behavior in learning activities, all of which contribute to their academic success [19,20].This implies that students with a high level of academic self-efficiency would develop autonomous learning strategies and innovative and creative behavior and lead to positive outcomes [21,22].

A growing body of studies has linked family cultural capital to improved cognitive skills in students [23,24]. Research from comparative cross-national settings, such as OECD and non-OECD countries/regions, is scarce. This paper first investigates the impacts of family cultural capital on the cognitive achievement of 15-year-old students, using large cross-national data from PISA 2022, which includes a sample from 71 nations and regions. It also explores the role of academic engagement and academic self-efficacy as mediators between these impacts. Importantly, the study fills the research gap by comparing the extent to which the influence of family cultural capital on students’ cognitive performance varies across OECD and non-OECD areas.

## Prior research

### Family cultural capital and student cognitive performance

For years, through the Cultural Reproduction Theory, Bourdieu popularized cultural capital as a multidimensional concept that pertains to knowledge, skills, education, values, and beliefs that individuals acquire through their upbringing and socialization within a particular culture production and reproduction [25,26]. Family cultural capital impacts the social, educational, and economic opportunities of family members, especially children [27]. Although Bourdieu conceptualized cultural capital as comprising three subdimensions: incorporated, institutionalized, and objectified, which enable the elite class to transmit and reproduce social class across generations [28,29], there remains a lack of consensus among scholars regarding its definitive indicators or golden standards [29,30]. The reason for this lack of consensus is the unclear boundaries between cultural capital and family-background variables, social practices, as well as the advancing digitization of education [31–33]. Contextually, family cultural capital refers to the general and interdisciplinary collection and the number of books from the home environment that contribute to children’s educational supports [34,35].

Scholars believe that family cultural capital plays a fundamental role in shaping children’s future prospects, because, it transmits or reinforces educational pedagogies and also significantly influences children’s socioemotional, economic, and job success [10,36]. For instance, the results of the Ciftci et al. [37] study demonstrated that the cultural capital of parents positively influences children’s likelihood to become elite scientists, when other studies have discovered the connection between cognitive performance and family cultural [38]. Meaning that, children from culturally privileged families tend to exhibit superior academic readiness, elevated educational goals, and perform better cognitively [39,40].

### Cultural capital and academic engagement

Academic engagement is defined as the extent to which an individual or group of people pay close attention to academic and learning-related tasks in order to achieve some academic goals [41]. Scholar has discovered conductive links between student responsiveness, academic learning environments in the family, and academic engagement [42]. Specifically, when students have some cultural capital heritage from parents, they tend to be emotionally, psychologically, and socially engaged by prioritizing academic meetings, virtual or face-to-face academic discussion, adopting a positive attitude, and actively participating in school activities [43]. Furthermore, students with a privilege cultural capital have the ability to develop cultural identity and a sense of belonging, enabling them to engage in high-level education [44]. Wenner also found that formal engagement in the scientific and math capital of families significantly influences children’s school engagement, their interest and aspirations, and their choice of science or math studies [42,45].

### Cultural capital and academic self-efficacy

Bandura’s social cognitive theory has emphasized that self-efficacy is a confidence individuals have about their resources, skills, and knowledge necessaries to achieve some expected outcomes [18,46]. In education, researchers claimed that students with a high amount of self-efficacy excelled academically [17,22]. This is due to the fact that when students have a high level of self-efficacy, they are more likely to develop a resilient, persistent, and challenging attitude, which in turn enables them to overcome academic obstacles [20,22]. Self-efficacy is then considered a pivotal catalyst that psychologically motivates students to establish ambitious goals and leads them to pursuing satisfactory academic success.

Past studies have revealed the role of family cultural capital in predicting academic self-efficacy [21,39,40]. It is noted that cultural background and educational aspiration of parents can influence student self-efficacy, behavioral control, and growth mindset can affect a student’s academic performance and career choices [47,48]. This is because when a student grow up within the environment with a great number of books with different categories at home and learning resources, they adopt the attitude directed toward privileged intellectual and human capital [49].

### Academic engagement and cognitive performance

Academic engagement refers to students’ active psychological and behavioral participation and investment in academic activities and learning processes, whereas disengagement refers to student disaffection or lack of engagement [50]. In education, academic engagement is known as a significant predictor and promotor of student general and specific academic success [43,51]. Educational theories such as expectancies for success have demonstrated that when individuals have confidence in their abilities and skills and aspire to excel academically, they become more engaged, and this engagement in turn contributes to their cognitive achievement [52]. Furthermore, scholars have underscored that students who are academically engaged exhibit prosocial behaviors such as regular attendance, completion of school tasks and assignments, active and collaborative discussions, and active classroom interaction [53]. According to Palos et al. [14], academic engagement also helps learners build their psychological capital, improve their critical thinking, academic resilience, and effective problem-solving attitudes, actively engage in knowledge that pertains to cognitive performance and anticipated employment [15]. Academic engagement positively correlate with positive school-family partnerships, fruitful teacher-student relations, peer relationships, teamwork, and the enhancement of self-efficacy and self-concept [13].

### Student academic self-efficacy and cognitive performance

Based on Bandura’s social cognitive theory, scholars have been investigating people’s developmental, social cognition, and sociocultural growth for some decades [54,55]. It is known that human self-efficacy, defined as the perception and confidence in one’s capabilities, drives individuals’ intrinsic motivation. Past studies have demonstrated that student self-efficacy is a significant predictor of their cognitive performance [55,56]. Researchers have noted that children’s language, mathematics and science self-efficacy profoundly influences their successful language learning and their mathematical or scientific abilities [57,58]. Furthermore, self-efficacy is considered to be the pivotal factor in fostering student socioemotional attitude and cognitive and behavioral motivation. Meaning that, the higher the level of student self-efficacy, the more it influences students’ psychological motivation and the higher their cognitive achievement [59].

### The present study

**Fig 1:**
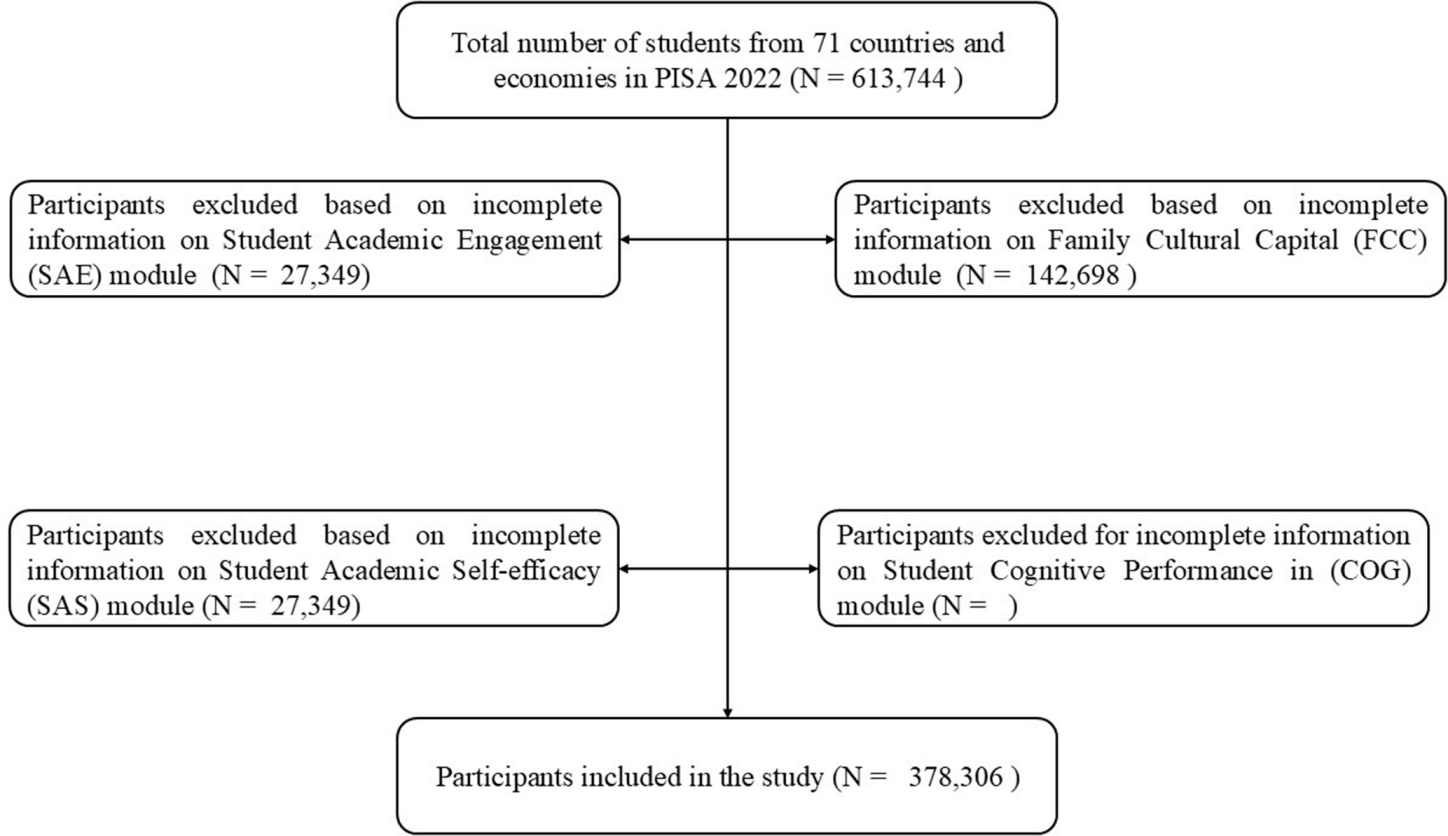
Flowchart of the excluded and included participants.

**Fig 2:**
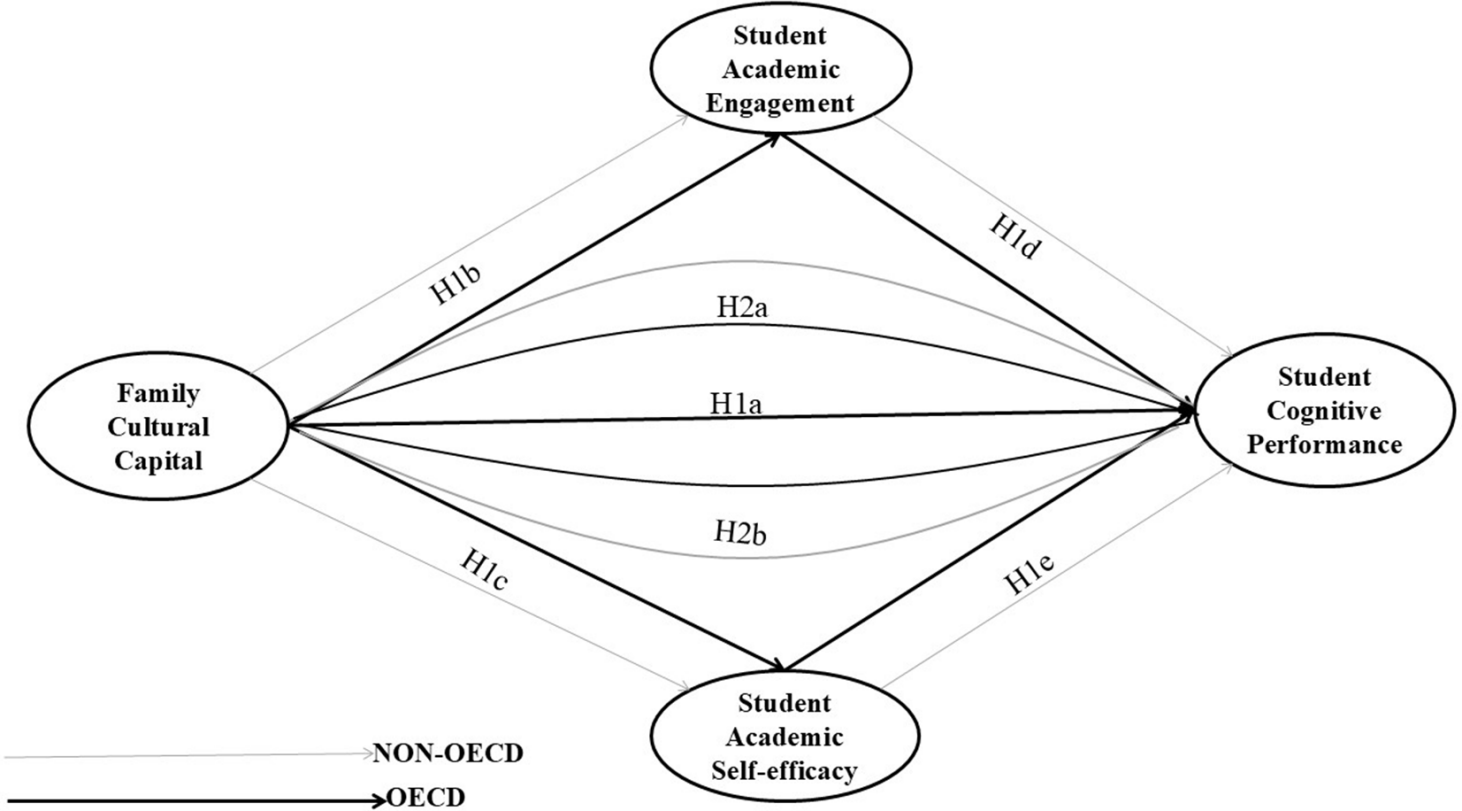
Hypothesis model.

This study examines the relationships between family cultural capital and student cognitive performance, focusing on the mediating effects of student academic engagement and academic self-efficacy in 33 OECD countries and 38 non-OECD nations/economies and comparing the effect sizes of these variables. The rationale behind this comparison is to gain insight into whether the effects of input variables on expected outputs differ between OECD and non-OECD countries. The study uses the PISA 2022 survey data to examine the relationships. After merging the PISA school data with student data, we excluded the non-valid responses due to the missing information. Fig 1 presents the flowchart of the excluded and included participants. Finally, we used only the valid sample to test two main hypotheses presented in Fig 2 below.

H1a: There is a direct positive correlation between family cultural capital and student cognitive performance in both OECD and non-OECD countries/economies, with a greater impact in OECD countries.

H1b: Family cultural capital is directly and positively associated with student academic engagement; the extent of this influence varies between OECD and non-OECD countries/districts.

H1c: There is a direct and positive association between family cultural capital and student academic self-efficacy across nations; however, the extent of this influence is likely to be lower in OECD nations.

H1d: We expect a positive direct correlation between student academic engagement and cognitive performance across different countries, but we expect this relation to be lower in OECD countries than in non-OECD countries.

H1e: There is a positive link between student academic self-efficacy and cognitive performance, but this relationship differs between OECD and non-OECD countries/regions.

H2a: Student academic engagement partially mediates the relationship between family cultural capital and student cognitive performance from several countries and economies; the mediation effects are expected to be lower in OECD than in non-OECD countries.

H2b: Student academic self-efficacy partially mediates the relationship between family cultural capital and student cognitive performance. We expect a greater mediating effect in non-OECD countries.

## Methods

### Participants

**Fig 3:**
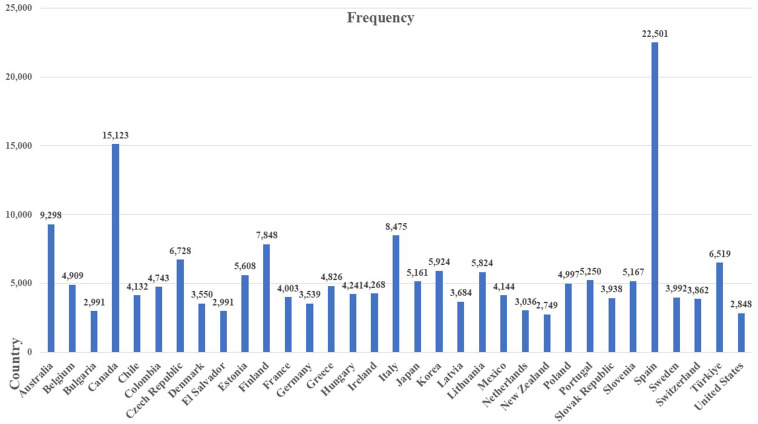
Included 33 OECD countries.

**Fig 4:**
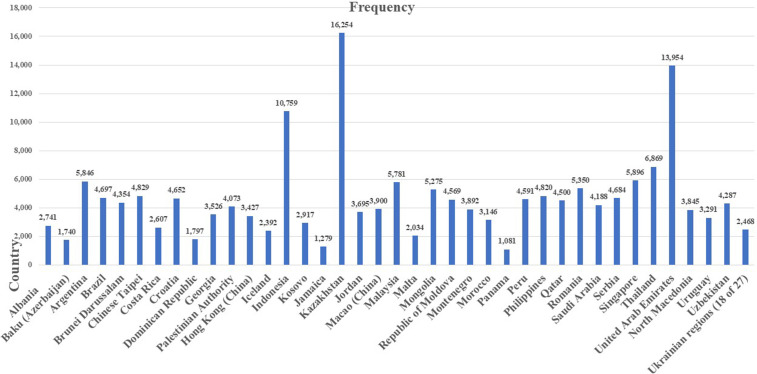
Included 38 non-OECD countries/economies.

The current study utilized large-scale data from PISA 2022, which included 378,306 15-year-old respondents from 33 OECD countries and 38 non-OECD nations/economies. The ethical consideration process for using PISA data is guaranteed by the OECD, who designed and coordinated the assessments to protect participants, including 15-year-old students, teachers, school principals, and parents/guardians. PISA data anonymizes personal identities and implements data security protocols for the participating countries/districts. Focusing on educational research and statistical comparability, the OECD provides detailed technical reports, sampling design processes, and transparent instruments at each round. In our sample, the lists of the included countries for both OECD and non-OECD counties/economies are presented in Fig 3 and Fig 4. In the total sample, each country’s proportion ranged from the lowest size with Panama (N = 1,081; 0.29%) to the highest size with Spain (N = 22,501; 5.95%). The average age in the PISA test is 15, and the sample mean is 1.48 (SD = 0.503). Regarding students’ gender, 51.06% of the participants from OECD countries were girls, compared to 52.95% in non- OECD countries.

### Variables

#### Dependent variable.

In the current study, the Item Response Theory (RIT) standardizes plausible values in math, reading, and science to measure the latent construct known as student cognitive performance. The study presents construct validity indices and standardized factor loadings for student cognitive performance. The Cronbach for cognitive performance includes 30 items is 0.994, indicating satisfactory construct validity. The Kaiser-Meyer-Olkin (KMO) sampling adequacy test score is 0.988, and the standardized factor loadings range from 0.90 to 0.93. This shows that the latent construct is valid in the real world.

#### Independent variable.

The PISA 2022 dataset’s index of home possessions across various book categories serves as the measurement for the constructed latent independent variable named family cultural capital, which consists of 8 items. PISA assessed students using a 5-point scale from 1 (none) to 5 (I don’t know). The question, “How many of the following types of books are in your home?” required respondents to select answers such as “Books to help with your schoolwork.” Cronbach’s alpha for the independent variable is 0.88, and the Kaiser-Meyer-Olkin (KMO) is 0.918; the standardized factor loadings range from 0.49 to 0.81, indicating that the latent construct is empirically valid.

In the extant literature, there has been a lack of consensus about the appropriate measures that can holistically capture the concept of cultural capital. For decades, although past studies proposed family participation in highbrow or other cultural activities to measure cultural capital, some scholars considered the definition to be too narrow [60,61]. Researchers have claimed that the conceptualization of cultural capital should not solely adhere to the dominant interpretations of highbrow or ‘technocratic” cultural capital, which are based on measures of ability, technical skills, competence, and school certificates [35,62]. Therefore, they suggest definitions that are oriented towards resource assistance, because they acknowledged that the technical skill conceptions should not be excluded from cultural capital discussion, but because there is no elaborated process through which “inherited cultural capital” contributes to next-generation educational outcomes, it can be seen as a supplementary resource that students may rely on for their academic success [63].

Our family cultural capital construct aligns with the existing literatures when focusing on the more tangible and examined print access educational resource that family possess to support children education [64,65].

#### Student academic engagement.

In this study, we build student academic engagement as a latent construct and test it using a six-item scale. School principals responded to eleven items in the PISA survey, indicating the extent to which the proposed factors at both the student and school levels hinder student learning in their institution [66]. Respondents’ responses on a four-point Likert scale ranged from (1) “not at all” to (4) “a lot.” The sample items are “Students skipping classes” and “Students not being attentive.” We reversed these items using the gen (1 = 4), (2 = 3), (3 = 2), and (4 = 1) recode command in Stata. With the recoded values, a score of 4 represents the highest, while a score of 1 represents the lowest. Cronbach’s alpha for the student academic engagement construct is 0.868, and the Kaiser-Meyer-Olkin (KMO) is 0.831; the standardized factor loadings range from 0.72 to 0.81, which is empirically favorable.

#### Student academic self-efficacy.

A nine-item scale measures the latent construct known as student academic self-efficacy, which is a second mediating variable in this study. The PISA 2022 survey asked students to indicate their level of agreement or disagreement with proposed statements about their confidence in their abilities and ambitions in math, reading, and science. For instance, students selected their responses based on an assertion like, “Mathematics is one of my favorite subjects” or “I want to do well in my science class.” Respondents’ answers on a four-point Likert scale ranged from (1) “strongly disagree” to (4) “strongly agree.” Student academic self-efficacy Cronbach’s alpha is 0.79, and the Kaiser-Meyer-Olkin (KMO) is 0.68; the standardized factor loadings fall between 0.66 and 0.88.

In this study, several variables were controlled as demographic background variables to avoid the confounding factors that could potentially interfere in the analysis. At the student level, the controlled variables include gender, age, and grade level. At the family level, economic, social, and cultural status (ESCS), immigration background (IMMIG), and mother’s level of education are measured by the International Standard Classification of Education (ISCED). In addition, school climate, school size (SCHSIZE), student-teacher ratio (STRATIO), and quality of teacher-student relation (RELATST) are controlled at the teacher and school level.

### Analytic Strategy

In the present study, we utilized STATA version 15.1 (Stata Corp LLC, College Station, TX) software to compute all the statistical analyses. We used a partial least squares structural equation model (PLS-SEM) to test the relationships between the predictor variables and the predicted outcomes. This model also assisted in determining the extent to which student academic engagement and academic self-efficacy act as mediators in these relationships.

Methodologically, PLS-SEM allows the researcher to statistically evaluate the extent to which empirical data confirms or declines the proposed hypotheses and theoretical predictions. We chose the PLS-SEM method for this study based on several advantages and due to its superiority and flexibility. First, for years, researchers in social sciences have recognized that PLS-SEM is a popular method for studying complex relationships between observables and latent variables [67–69]. The second reason for choosing PLS-SEM is the predictive objectives in this research. When the main goals of the structural equation model are to predict and explain specific outcomes, researchers suggest using PLS-SEM [70]. The third rationale for using PLS-SEM is its ability to measure the model effectively. With PLS-SEM, researchers can easily compute the formative constructs, enhance the explanatory value, and reduce the error term and measurement error in the construct by treating the explanatory and outcome variables as separate latent constructs [71]. Additionally, it has been shown that using PLS-SEM for data analysis is more effective than traditional methods like linear regression or hierarchical linear modeling because it allows for the simultaneous estimation of both direct and indirect relationships, which reduces errors across many paths [72]. This is because estimating both direct and indirect connections at the same time reduces error terms for many paths when using SEM [73,74]. The fourth reason is about the sample size. Compared to covariance-based SEM (CB-SEM), which uses all the data, PLS-SEM is suitable for smaller sample sizes, can handle non-normally distributed data, and is robust to cope with skewness and model complexity [67,75]. All these characteristics make it appropriate for analyzing our model with partial sample size data from PISA 2022. The study tested the goodness-of-fit and evaluated mediation effects using three independent statistical tests: Delta, Sobel, and Monte Carlo, employing 5000 bootstraps. In Table 1, we reported the Constructs reliabilities and factor loading of all the key variables.

**Table 1:** Constructs reliabilities and factor loading.

Constructs	Items	Mean	SD	Skew	Kurtosis	Factor loading	Cronbach’s α
Student cognitive performance	30	443.194	319.562	0.220	2.637	0.908- 0.936	0.994
Family cultural capital	8	2.195	1.099	1.206	3.855	0.667- 0.813	0.868
Student academic engagement	6	2.447	0.803	0.080	2.533	0.725- 0.816	0.868
Student academic self-efficacy	9	2.447	0.803	0.080	2.533	0.663- 0.883	0.795

## Results

### Descriptive statistics

In Table 2, we present the descriptive results of our sample. In terms of demographic characteristics, students in grades 7–13 in OECD countries have a mean age of 15.916 and a standard deviation (SD = .13.970), while students in grades 7–12 in non-OECD countries and districts have a mean of 15.934 and a standard deviation (SD = .13.074). Regarding students’ gender, 51.06% of the participants from OECD countries were girls, compared to 52.95% in non- OECD countries. In terms of family educational level measured by the International Standard Classification of Education (ISCED), respondents reported that 82.89% of mothers have completed ISCED ≥ 3 and 15.05% have ISCED ≤ 2 in OECD countries versus 74.94% and 22.4%, respectively, in non-OECD countries.

**Table 2:** Demographic characteristics.

Variables		OECD				Non-OECD			
		n	%	Mean	SD	n	%	Mean	SD
Age		186,869		15.916	.276	180,006		15.934	.247
Gender	Female	95,412	51.06			95,317	52.95		
	Male	91,403	48.91			84,689	47.05		
Socio economic status		186,869		.534	6.963	180,006		1.237	12.633
Immigration background		186,869		3.535	1.592	–		1.389	1.366
Student cognitive performance		186,869		494.290	86.351	180,006		434.558	90.268
Grade level		(7-13)		11.938	13.970	(7-12)		11.171	11.074
Mother education	ISCED ≤ 2	28,144	15,05			40,329	22,4		
	ISCED ≥ 3	154,903	82,89			134,910	74,94		
School size		186,869		1.16e + 07	3.20e + 07	180,006		251	1.57e + 07

### Direct and mediating analysis

The PLS-SEM model fits well, as shown by the model fit statistics: Chi-square test, $\chi_{\text{1}\text{3}\text{2}\text{0}}^{\text{2}}$ = 2,750.3 (df = 180, *p* < 0.001); Akaike’s information criterion (AIC) = 8.286e + 07; Bayesian information criterion (BIC) = 8.286e + 0; Comparative Fit Index (CFI) = 0.980; Tucker-Lewis index (TLI) = 0.970; Standardized Root Mean Square Residual (SRMR) = 0.065; Root Mean Square Error of Approximation (RMSEA) = 0,035, and coefficient of determination (CD) = 0.92 [76,77]. The recorded values of goodness-of-fit test indicated an adequate model fit because they fall within the standardized limits the application can enhance the effectiveness when evaluating SEM model [78].

**Fig 5:**
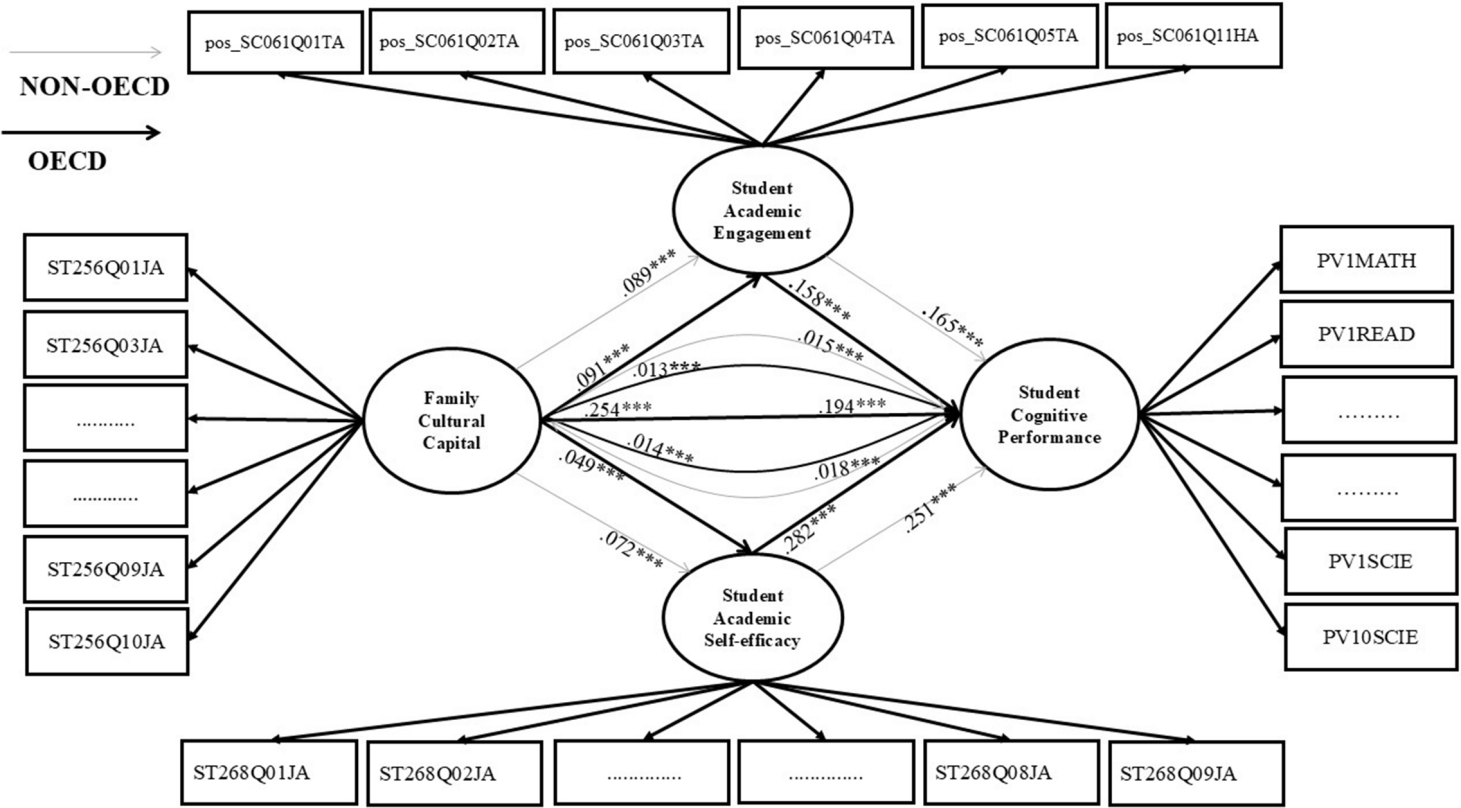
The direct and indirect effects results.

Furthermore, before running the PLS-SEM test, we employed the Pearson Correlation Coefficient Matrix to check for multicollinearity between the used variables. Table 3 displays the relationships among key variables related to students’ academic development. The generally low correlations among variables indicate that each represents a distinct construct, reducing concerns about multicollinearity. These variables are well-suited for the study because they reflect a comprehensive view of the factors influencing academic outcomes. Finally, we conducted a model test on the direct and indirect relationships of the PLS-SEM, and Table 4 and Fig 5 display the results.

**Table 3:** Substantive covariates Correlations.

Covariate	1	2	3	4	5	6	7	8	9	10	11	12	13
1-FCC	1.0000												
2-SAE	0.0794	1.0000											
3-SAS	0.0455	0.0510	1.0000										
4-COG	0.2479	0.1747	0.0510	1.0000									
5-Gender	0.0832	−0.0352	0.1747	0.2949	1.0000								
6-Age	0.0293	0.0170	−0.0352	−0.0088	0.0052	1.0000							
7-Grade level	−0.0244	−0.0356	0.0170	−0.0171	0.0088	0.0208	1.0000						
8-ESCS	0.0667	0.0049	−0.0356	−0.0149	−0.0200	−0.0083	−0.0081	1.0000					
9-Mother education	−0.0031	−0.0262	0.0049	−0.0048	0.0186	−0.0053	0.0051	0.4627	1.0000				
10-IMMIG	−0.0652	0.0257	−0.0262	−0.0288	−0.0772	−0.0903	−0.0078	0.0495	0.0513	1.0000			
11-SCHSIZE	−0.0136	−0.1501	0.0257	−0.0258	−0.0150	0.0279	−0.0038	0.0241	0.0096	0.0513	1.0000		
12-STRATIO	−0.0169	−0.1250	−0.1501	0.0781	0.0489	0.0423	−0.0141	0.0111	−0.0164	0.0096	0.8301	1.0000	
13-RELATST	−0.0162	−0.1506	−0.1250	0.0748	0.0377	0.0870	−0.0074	0.0252	−0.0113	−0.0164	0.8001	0.7645	1.0000

**Notes:** FCC = Family Cultural Capital, SAE = Student Academic Engagement, SAS = Student Academic Self-Efficacy, COG = Student Cognitive Performance, IMMIG = Immigration background, ESCS = economic, social, and cultural status, SCHSIZE = school size, STRATIO = student-teacher ratio, RELATST = quality of the teacher-student relationship.

**Table 4:** Structural equation model results for mediation analysis.

Independent Variable		Dependent Variable	Std. coefficient	Z	p-value	[95% Conf. Interval]
Direct effects (non-OECD)	FCC	COG	0.194***	83.81	< 0.001	[0.190, 0.190]
	FCC	SAE	0.089***	33.60	< 0.001	[0.084, 0.094]
	FCC	SAS	0.072***	27.06	< 0.001	[0.067, 0.077]
	SAE	COG	0.165***	71.86	< 0.001	[0.161, 0.170]
	SAS	COG	0.251***	110.59	< 0.001	[0.247, 0.256]
	**SAE**	**SAS**	0.063***	**24.06**	**< 0.001**	**[0.058, 0.068]**
Direct effects (OECD)	FCC	COG	0.254***	114.71	< 0.001	[0.250, 0.259]
	FCC	SAE	0.091***	34.30	< 0.001	[0.085, 0.095]
	FCC	SAS	0.049***	18.75	< 0.001	[0.044, 0.054]
	SAE	COG	0.158***	66.66	< 0.001	[0.153, 0.162]
	SAS	COG	0.282***	124.73	< 0.001	[0.277, 0.286]
	**SAE**	**SAS**	0.095***	**35.66**	**< 0.001**	**[0.090, 0.100]**
Indirect effect (non-OECD)	FCC → SAE → COG		0.015***	30.707	< 0.001	[0.014, 0.016]
	FCC → SAS → COG		0.018***	26.481	< 0.001	[0.017, 0.020]
Indirect effect (OECD)	FCC → SAE → COG		0.013***	30.800	< 0.001	[0.013, 0.015]
	FCC → SAS → COG		0.014***	18.653	< 0.001	[0.013, 0.015]
Total effect (non-OECD)	FCC → COG		0.227			
Total effect (OECD)	FCC → COG		0.281			

Model fit matrix$:\;\chi_{\text{1}\text{3}\text{2}\text{0}}^{\text{2}}$ = 2,750.3 (df = 180, *p* < 0.001). CFI = 0.980, TLI = 0.970, RMSEA = 0.035, SRMR = 0.065, CD = 0.92, AIC = 8.286e + 07, BIC = 8.286e + 07. FCC: Family cultural capital, SAE: Student academic engagement, SAS: Student academic self-efficacy, COG: Student cognitive performance, *p < 0.01, ***p < 0.001.

For direct effects, after controlling demographic background variables at the student, family, school, and teacher levels, we found that every standard deviation increase in family cultural capital is associated to an improvement in student cognitive performance in OECD countries (std. β = 0.254, *p* < 0.001) and non-OECD nations (std. β = 0.194, *p* < 0.001), academic engagement in OECD countries (std. β = 0.091, *p* < 0.001) and non-OECD nations (std. β = 0.089, *p* < 0.001), and academic self-efficacy in OECD countries (std. β = 0.049, *p* < 0.001) and non-OECD countries (β = 0.072, *p* < 0.001). These findings support and confirm H1a, H1b, and H1c. In addition, positive relationships exist between student academic engagement and cognitive performance in OECD and non-OECD countries (β = 0.158, *p* < 0.001; β = 0.165, *p* < 0.001, respectively), and academic self-efficacy and cognitive performance in OECD countries (β = 0.282, *p* < 0.001) and non-OECD countries (β = 0.251, *p* < 0.001), which also support and confirm H1d and H1e.

For the indirect effects in both OECD and non-OECD countries, student academic engagement plays a crucial mediating role in the positive links between family cultural capital and student cognitive performance (β = 0.013, **p* *< 0.001; β = 0.015, **p* *< 0.001, respectively). The relationship is also partly and significantly mediated by academic self-efficacy (β = 0.014, *p* < 0.001; β = 0.018, *p* < 0.001) for OECD and non-OECD countries. These results reveal that academic engagement and academic self-efficacy mediate the indirect effect of family cultural capital on student cognitive performance, further confirm H2a and H2b. In OECD nations, academic self-efficacy mediates about 5% of the relationships, while academic engagement mediates 4%. In non-OECD countries, academic self-efficacy mediates about 6% of relationships and academic engagement about 8%; these findings empirically validate H2a and H2b.

## Discussion

The purpose of the present study is to first investigate the multidimensional relationships between family cultural capital and student cognitive performance through the mediation of psychological and behavioral variables (academic engagement and academic self-efficacy). Secondly, the study aims to compare the differences in effect size between OECD and non-OECD countries. Researchers have demonstrated that having books at home, conceptualized as a “home library,” “scholarly culture,” or sometimes “scholarly family,” is a form of cultural capital that plays a fundamental role in predicting student cognitive performance globally, regardless of educational systems, income levels, historical and cultural backgrounds, sociopolitical, and the dominant ideology [35,79]. However, there is a scarcity of recent literature using large cross-national datasets to investigate this intrinsic and complex relationship from a comparative perspective. To fill the gap, the current study leverages data from PISA 2022 with a sample of 71 countries and uses PLS-SEM to estimate the relationship based on two main hypotheses.

PLS-SEM direct results revealed a direct, positive link between family cultural capital and student cognitive performance, with a stronger effect size in OECD countries than in non-OECD countries. Second, there is a direct positive connection between family cultural capital and student academic engagement in both OECD and non-OECD countries. However, the effect is stronger in the OECD zone compared to non-OECD counterparties. Thirdly, we found that family cultural capital positively predicts student academic self-efficacy, but the gains are more in non-OECD countries than in OECD countries. Fourth, a positive relationship exists between student academic engagement and cognitive performance in the compared countries, and different levels of impact are observed in OECD and non-OECD countries. Fifth, there is a positive relationship between student academic self-efficacy and student cognitive performance. However, student academic self-efficacy promotes more cognitive outcomes for 15-year-old OECD students than for their non-OECD peers when comparing the effect size between OECD and non-OECD countries.

Furthermore, the links between the predictor variable and the predicted outcomes were partially mediated by student academic engagement and academic self-efficacy, also with the effect size varying between OECD and non-OECD countries. All the direct and indirect findings are consistent with prior studies [15,20,54,80]. Below, we will discuss these key findings from structured institutions, societies, families, and schools, followed by teacher and student characteristics.

### Theoretical implications

#### Cultural capital and cognitive performance.

Theoretically, the study proposed a model that aligns with previous research on Bourdieu’s cultural capital theory when considered bookish families as elite closure or privileged class ownerships [81,82]. This theoretical model asserts that, despite being material goods, books differ from other physical capital accumulated goods because they contain words, languages, knowledge, beliefs, and secrets, all of which play a crucial role in shaping students’ cognitive skills across various contexts [26,83].

Interestingly, comparing OECD countries with non-OECD countries reveals some disparities, even though all results are positively associated with student cognitive performance. Specifically, OECD countries, which are predominantly high-income countries, have political systems that are Western, democratic, and capitalistic, and they have a rich history of elite education [84,85]. Families possessing a large quantity of high-quality, well-structured, and canonized books at home not only symbolize their social, political, and economic level; book-rich families, compared to bookless ones, have access to history, coded language, science, and an imaginative world capable of molding student cognitive performance [86]. Moreover, students with wealth and highly educated mothers who are more likely to be involved in school decision-making and the educational system prioritize the integration of cultural knowledge and ecological environments, may be typically exposed to the same categories of books at home and in school, and are being exposed to advanced knowledge such as artificial intelligence that shape and enhance their cognitive skills [87].

In non-OECD countries, which are predominantly middle- and low-income countries, some with instable political and cultural systems, education systems may have limited opportunities of practices, disconnect between school curricula and student home daily life, the lack of the extra supportive ecosystem, differences in educational priorities, parents have low levels of education with less positive attitudes about books and reading for pleasure, and the teaching environment [42,88]. Meaning that even families have a large number of books at home, they may not be used effectively to enhance student cognitive outcomes. Additionally, parents with a high cultural capital may choose to engage their children in vocational skills or immediate learning outcomes needed by family, such as learning other languages, rather than focusing on enhancing their cognitive abilities [89].

#### Family cultural capital and academic engagement.

The result also confirms the H1b, which suggests that an increase in family cultural capital has a greater effect on student academic engagement in OECD countries compared to non-OECD countries. In detail, families in OECD countries focus on collecting books that help students build knowledge and skills and directly support their academic interest and engagement [90]. Scholars agree that providing a learning environment with a variety of books and educational materials, which supports intra- and extracurricular programs to foster curiosity and interest, is a crucial factor for students’ motivation for academic success [35,44,91,92]. Furthermore, OECD educational systems emphasized and engaged students in reading programs, encouraged family involvement to discuss books with kids, promoted independent learning decisions, and implemented personalized learning plans that emphasized family cultural capital and enhanced academic engagement [93].

In contrast, some non-OECD countries with poorly resourced education systems, high student-teacher ratios, less qualified teaching staffs, a lack of infrastructure like public resources that leads the weight of education only to family, and having books may not play a motivator role in engagement [94]. Moreover, parents may have limited categorized and structured books that are related to the school curriculum, especially when books become a serious source of financial investment, difficult to access, or a luxury good. Furthermore, due to issues arising from social norms, values, tradition, and beliefs, some conservative families may restrict their book collection to books related to religious or local cultures, thereby limiting their children’s exposure to challenging and classical works. This lack of intellectual and cognitive stimulation may not directly enhance student engagement, but instead lead to disengagement [27].

#### Academic engagement and cognitive performance.

As hypothesis H1d indicates, student academic engagement is also a significant promoter for student cognitive performance across both OECD and non-OECD countries. However, the effect size is relatively low in OECD countries. OECD countries that prioritize equitable access to well-equipped schools, digitalized infrastructure, high-tech and technology-based lessons, and professional teacher training continue to foster cognitive development [95]. But this effect is less significant in boosting cognitive performance, because within the education systems that prioritize higher achievement, students already benefit from different sources and generally perform better cognitively, though their effort may still contribute to positive enhancement [96,97]. Also, in an educational system where high-stakes exams or school attainment is not a unique avenue to economic mobility due to more established social safety nets, it may create an atmosphere of nonurgency for the influence of engagement on cognitive performance.

However, students in non-OECD countries often encounter systemic educational disparities; their academic engagement significantly contributes to improving their cognitive performance, particularly when they are aware of their lack of other supports [15]. Students in non-OECD countries may engage themselves behaviorally, emotionally, and cognitively, and they may seek additional learning through self-study or extra effort as a form of compensation [39,82]. This engagement can enhance cognitive performance when they and their societies view educational success as a crucial pathway to social mobility and future job opportunities. The fear for economic stability in the competitive academic environment and social and family pressure may create an atmosphere of urgency, and student engagement becomes a pivotal factor for cognitive performance [98].

#### Family cultural capital and student’s academic self-efficacy.

Family cultural capital significantly predicts students’ academic self-efficacy, with the OECD zones showing the lowest effect size. Most OECD countries have advantages in implementing policies aimed at reducing educational inequalities, such as publicly funding schools for the disadvantaged and students with special needs [99]. Further, the public supports interventions, such as professional development or family involvement, that enable students from different backgrounds to develop independent self-efficacy rather than being dependent on family cultural capital [100,101]. Moreover, their systems offer ample resources such as teaching materials, which often mirror those found in students’ homes, thereby diminishing the influence of family books on student self-efficacy. Studies in the past have observed that certain cultures and societies prioritize individualism, stressing the importance of individual accomplishment [102–104]. They perceive academic self-efficacy as a personal duty, while classroom instruction prioritizes the empowerment of individual values.

In many non-OECD countries, on the other hand, where schools may not have as much access to technology, there is less of a link between public resources like book collections and family scholarly culture. This means that families’ academic goals and the number and quality of books they own become more important for bridging the gaps between producing and reproducing human capital, which has a direct effect on the academic self-efficacy of future generations. This means that families with high cultural capital in non-OECD countries compensate for the public gaps by exposing their children to a stimulating environment at home, where there are plenty of multidisciplinary books, which reinforces their psychological motivation, confidence, and belief in their ability to handle academic work [105].

### Academic self-efficacy and cognitive performance

This study has discovered the well-established link between student cognitive performance and their academic self-efficacy in OECD and non-OECD countries that was theoretically meaningful and consistent with past studies [58,106]. Comparatively, the benefit is greater in OECD countries. Specifically, OECD education systems are well-resourced, allowing learners to empirically test their confidence through project-based learning, critical thinking exercises, and real-world problem-solving puzzles [102]. These activities contribute to the development of non-cognitive skills such as growth mindset and self-efficacy, psychological well-being, and autonomy and independence in learning choices, all of which enhance cognitive outcomes. The impact of academic self-efficacy is greater in OECD zones because their extracurricular activities, tutoring and counselling boards, professional teaching and feedback, constructive critics, customized instructions, and learning contents also allow children to have a high level of confidence, all leading to better academic self-efficacy and metacognition outcomes [57,107].

In non-OECD countries, students’ confidence in their academic abilities, coupled with psychological motivation, motivates them to adopt positive behaviors and strategies efficiently, which in turn leads to positive cognitive outcomes [108]. Students who are confident in their academic abilities are more likely to be focused and have an open and curious mind [109]. This can lead to general or specific cognitive outcomes like doing well on tests and solving math problems [56,110]. However, according to human ecology theory, skills gain is still lower than in OECD zones because of things like fewer educational facilities and bigger social environments [111].

### Mediating role academic engagement and academic self-efficacy

According to our predictions, family cultural capital can be linked to student cognitive performance through the enhancement of academic engagement, with the mediating effect expected to be less pronounced in OECD countries. This implies that the perception of family cultural capital varies between OECD and non-OECD countries, as cultural capital in some countries encompasses more than just having books at home. Further, non-OECD families used to fill a gap that schools cannot address; though there are still limited resources for education, having books at home in these nations stimulates engagement, which in turn plays an essential mediating role between family cultural capital and cognitive outcomes. Family aspirations for academic success and their investment led students to strive and engage academically and exercise their agency, which increased cognitive performance [57,107].

Student academic self-efficacy also plays a partial mediating role between the input and output variables link, albeit with a weaker effect in OECD countries. OECD countries, as previously mentioned, offer family-school-community-based environments, where the interactions among these trial factors often directly influence students’ cognitive performance. Further, well-resourced schools located in urban areas have kindergarten or preschool education preparation, focusing on emotional well-being and adaptive linguistic environments, all of which lessen the mediating role of academic self-efficacy in improving cognitive performance, though the effect is positive [89,112]. Nevertheless, in some non-OECD countries, some factors like poverty, most schools being in rural areas, other regions like Ukraine with wars, students lacking a safe learning environment, and there being a risk of never attending school, or students may study with hunger or food insecurity [85,113]. These students receive instruction in various languages; the presence of books in their families not only significantly enhances their cognitive and non-cognitive skills, but also serves as an important mediator between the variables.

### Practical implications

This study identifies several factors and suggestions necessary for educational changes. First, the proposed comparative theoretical model suggests that policymakers should improve students’ cognitive abilities, academic engagement, and academic self-efficacy through more than just diversifying or collecting books at home. It is crucial for decision-makers to update their theoretical knowledge, determine the necessary capital for each educational system, and devise effective policies to address it. Additionally, they should take into account whether children are born and raised in low-income, middle-income, or high-income countries and whether these children belong to low-income, middle-income, or high-income families [62,114].

In addition, because family cultural capital has an indirect effect on expected results and varies across countries, educational stakeholders should put an emphasis on both building family cultural capital and improving student academic engagement and self-efficacy through well-thought-out reforms and practices. Additionally, any programs aimed at enhancing students’ cognitive skills through the use of predictors and predicted variables should first identify “what works and what does not” in order to bridge the achievement gap between privileged classes and less fortunate groups. This will stop inequality in society, which can lead to democratic education.

## Conclusion

This study broadens our understanding of the significant role of family cultural capital in developing student cognitive performance, as well as the mediating effects of student academic engagement and academic self-efficacy. Our findings demonstrate a direct correlation between family cultural capital and student cognitive performance, academic engagement, and academic self-efficacy, albeit with varying effect sizes across OECD and non-OECD countries. The two mediator factors which confirm the theoretical model partially mediate the link between dependent and independent variables. The study contributes significantly to the field by determining the impact of cultural capital theory on student educational practices and providing practical and useful recommendations. It could help promote social equality by looking at how family book collections, resources, cultural values, and the home environment affect a student’s cognitive outcomes, their motivation, and their confidence to learn from different countries and regions. Our research suggests the need for interventions and reforms that target not only family cultural capital but also student psychological and behavioral factors, taking into account the economic and sociocultural contexts of the learners in order to promote equity and improve educational outcomes across diverse countries and economies.

## Limitations and suggestions

This study’s contributions are noteworthy; it is important to acknowledge some limitations. Firstly, future researchers should investigate these complex relationships between the factors by incorporating longitudinal and ethnographic studies to draw a robust conclusion and deepen our knowledge. Secondly, the PISA data focused solely on 15-year-old students; the correlations between the explanatory factors and the observed outcomes could vary depending on the age group or test conditions. To broaden our understanding, future studies can take into account the low or high range of age groups when investigating the pathways. Thirdly, the study solely examined the relationship between family cultural capital and student academic performance, with student academic engagement and academic self-efficacy acting as mediators. Given the complexity of these relationships, we can enhance our understanding by exploring additional mediating factors such as instructional practices and qualities, teacher-student communication, and student psychological capital. Fourthly, while family cultural capital is a broad proximal environmental factor that shapes children’s development, its effect on key outcomes based on home book possession makes the interpretation of the results more cautious. Although our hypothesis offers valuable comparative insights into the PLS-SEM results, future research should incorporate statistical tests to determine whether the differences are statistically significant, thereby enhancing our understanding of evidence-based cross-group differences. All these limitations should be considered when interpreting the findings.

## Supporting information

S1 Dataset Data from Student and parent questionnaire. (XLS)

S2 Dataset Data from School questionnaire. (XLS)
